# Synergistic effects of hyperbaric oxygen therapy combined with MPNFS nursing model on neurological recovery and recurrence prevention after chronic subdural hematoma surgery

**DOI:** 10.3389/fneur.2025.1642148

**Published:** 2025-09-09

**Authors:** Ying Xie, Dongmei Yang, Ting Jiang, Hongdi Liu, Yan Gao

**Affiliations:** Department of Neurosurgery, Changde Hospital, Xiangya School of Medicine, Central South University (The First People’s Hospital of Changde City), Changde, China

**Keywords:** chronic subdural hematoma, hyperbaric oxygen therapy, MPNFS nursing model, neurological recovery, recurrence prediction

## Abstract

**Background:**

Chronic subdural hematoma (CSDH) has high postoperative recurrence rates. This study investigates the effects of hyperbaric oxygen therapy (HBOT) combined with Medical-Psychosocial-Nursing Functional Support (MPNFS) on functional recovery and recurrence prevention in CSDH patients, and establishes a recurrence prediction model.

**Methods:**

A total of 184 CSDH patients undergoing burr hole drainage were randomized into a control group and an observation group (HBOT + MPNFS). Neurological (NIHSS), motor (Fugl-Meyer Assessment), and quality-of-life (SF-36) outcomes were assessed preoperatively and at 1-month postoperatively. Complications and 6-month recurrence rates were recorded. Univariate/multivariate logistic regression identified recurrence risk factors, with ROC analysis evaluating predictive accuracy.

**Results:**

The observation group showed superior 1-month outcomes: lower NIHSS scores (*t* = 4.94, *p* < 0.001), higher FMA and SF-36 scores (*p* < 0.01). Complication and recurrence rates were significantly reduced (*p* < 0.05). Independent recurrence predictors included brain atrophy (OR = 2.877), poor brain reexpansion (OR = 3.165), preoperative hematoma width ≥ 20 mm (OR = 2.782), and absence of combined intervention (OR = 2.842). The multifactorial model achieved an AUC of 0.7862, indicating robust predictive efficacy.

**Conclusion:**

Hyperbaric oxygen therapy combined with MPNFS enhances neurological/motor recovery, improves quality of life, and reduces complications/recurrence in postoperative CSDH patients.

## 1 Introduction

Chronic subdural hematoma (CSDH), a prevalent intracranial disorder among older adults, develops predominantly following minor head trauma and manifests with insidious onset and progressive neurological deterioration (1). Against the backdrop of global population aging, CSDH incidence has risen annually, now representing a leading cause of acquired neurological disability in elderly neurosurgical patients (2, 3). Epidemiological reports indicate an annual incidence of 58 cases per 100,000 older adults, with postoperative recurrence rates persisting at 5–30%, severely impeding neurological recovery, compromising quality of life (QoL), and escalating healthcare burdens and resource consumption (4, 5). Although burr hole drainage remains the cornerstone of minimally invasive management, persistent challenges including unpredictable recurrence rates and suboptimal functional outcomes underscore the need for optimized therapeutic strategies (6, 7). Consequently, developing effective postoperative interventions to facilitate neural reconstruction, enhance rehabilitation efficacy, and mitigate recurrence risk has become a pivotal research frontier in contemporary CSDH management.

Emerging as a promising adjunctive therapy in neurorehabilitation, hyperbaric oxygen therapy (HBOT) has been increasingly integrated into the management of central nervous system pathologies. In CSDH populations, HBOT has demonstrated multifaceted therapeutic benefits, including enhancement of cerebral oxygenation, mitigation of brain edema and inflammation, and promotion of neural repair, collectively contributing to improved neurological recovery (8, 9). Complementing these physiological interventions, perioperative nursing care play a critical role in optimizing functional outcomes. Conventional nursing paradigms, focusing on basic care and health education, often fails to address the multidimensional needs of CSDH patients, particularly regarding cognitive, motor, emotional, and behavioral rehabilitation.

The Medical-Psychosocial-Nursing Functional Support (MPNFS) nursing model, encompassing Medication, Psychological intervention, Nursing, Family care, and Social support, represents an innovative integrative care philosophy oriented toward functional recovery. It emphasizes systematic, goal-directed interventions during the perioperative period to promote neural rehabilitation, psychological adjustment, and restoration of activities of daily living (ADL) (10). Tailored to patients’ clinical characteristics and recovery stages, this model prioritizes synergistic effects among neurorehabilitation, cognitive enhancement, functional training, and emotional regulation, demonstrating high adaptability and practicality. Emerging research has shown that MPNFS-based comprehensive nursing significantly improves functional independence and life satisfaction (11). However, its application in CSDH rehabilitation remains underexplored, with particular paucity in evidence regarding its combinatorial effects with HBOT, which is a critical gap this study seeks to address through multidimensional outcome analysis.

Additionally, the intricate pathogenesis of CSDH recurrence, coupled with pronounced interindividual variability, has confined most prior studies to univariate risk factor analyses, thereby overlooking the need for integrated multivariate frameworks. This methodological gap has posed a critical barrier to the development of precise, predictive recurrence models, impeding the implementation of proactive postoperative management strategies. Therefore, the construction of a scientifically robust risk prediction tool, paired with rigorous evaluation of its discriminative accuracy, remains essential for translating evidence into personalized, targeted interventions.

Addressing these unmet needs, this prospective randomized controlled study enrolls CSDH patients undergoing burr hole drainage to investigate the combined effects of HBOT and MPNFS, adjunctive to standard perioperative care, on neurological recovery, motor function reconstruction, and QoL. Furthermore, multivariate logistic regression analysis is adopted to identify independent recurrence risk factors, and receiver operating characteristic (ROC) curves are plotted to assess the predictive model’s efficacy, aiming to provide clinical evidence for refined postoperative CSDH management.

## 2 Materials and methods

### 2.1 Study population

In this prospective study, a total of 184 consecutive CSDH patients undergoing burr hole drainage were enrolled from the Critical Care Medicine Department in our hospital between September 2022 and December 2024. Participants were randomly allocated into two groups (*n* = 92 each) using a random number table. The observation group comprised 61 males and 31 females with an average age of 61.73 ± 4.77 years, while the control group included 67 males and 25 females averaging 61.17 ± 4.52 years. Written informed consent was obtained from all patients or their legal guardians, and the study protocol received ethical approval from the Ethics Committee of our hospital.

Inclusion criteria: confirmed CSDH with subsequent burr hole drainage (12); complete clinical records; and signed informed consent from patients and families.

Exclusion criteria: severe cardiopulmonary comorbidities; absolute contraindications to HBOT (untreated pneumothorax, history of epilepsy); or poor treatment compliance.

### 2.2 Intervention protocols

#### 2.2.1 Hyperbaric oxygen therapy

Both groups received hyperbaric oxygen therapy (HBOT) postoperatively. Treatment began 48 h after extubation using an NG240-570A hyperbaric oxygen chamber at a pressure of 0.25 MPa. After pressurization and stabilization, patients inhaled oxygen for 60 min, maintained stable pressure for another 60 min, underwent decompression for 15 min, and rested for 10 min before receiving a second 60-min oxygen inhalation session. The treatment was administered once daily for four consecutive weeks.

#### 2.2.2 Control group: routine nursing care

Patients in the control group remained hospitalized throughout the treatment period, with an average length of stay comparable to that of the intervention group (approximately 4 weeks). In addition to HBOT initiated 48 h after surgery, patients received standard perioperative nursing care, which included assistance with preoperative examinations, vital sign monitoring, preoperative dietary guidance, and intraoperative support. Postoperative care included condition monitoring, complication prevention, drainage tube management, and basic nursing interventions.

#### 2.2.3 Observation group: HBOT combined with MPNFS model-based nursing intervention

On the basis of routine nursing care, the observation group received an additional nursing intervention based on the MPNFS (Medical–Psychosocial–Nursing Functional Support) model.

Overview of the MPNFS Model: The MPNFS model is a patient-centered, structured, and multidimensional nursing intervention framework. It consists of four core modules: medical management, psychosocial support, functional rehabilitation guidance, and lifestyle regulation. Designed to facilitate postoperative recovery, improve quality of life, and support social reintegration, the model is adapted from stroke unit-based comprehensive care. It emphasizes interdisciplinary collaboration, individualized rehabilitation planning, and active involvement of both patients and their families. Compared to routine care, its distinctive features include: (1) structured psychological interventions and family engagement; (2) integrated physical rehabilitation and daily living training; and (3) continuous post-discharge health management.

Specific Components of the MPNFS-Based Intervention: ① Health education and psychological support: Disease and postoperative rehabilitation knowledge were popularized to patients and their families through multimedia education (videos, lectures). Psychological interventions were carried out according to individual characteristics, encouraging patients to express emotions and build rehabilitation confidence, and emphasizing family support to create a positive atmosphere (in contrast to the one-way health education in routine care). ② Posture and activity guidance: patients were instructed to lie on the affected side or in the supine position. Nursing staff assisted with turning and toileting, and encouraged early ambulation. Effective coughing techniques were taught, low-flow oxygen inhalation was provided when necessary, and pressure-relieving pads were used to prevent pressure ulcers. ③ Drainage management: Customized fluid replacement plans were formulated. Drainage tubes were kept unobstructed by preventing kinking or external compression. Drainage fluid was closely monitored, and the initial drainage height was positioned 10–15 cm above the hematoma cavity, followed by a gradual reduction of 5 cm every 5 h. Drainage bags were replaced under strict aseptic techniques to minimize infection risk. ④ Dietary intervention: Enteral nutrition was initiated as early as possible postoperatively. After regaining consciousness, patients first received a trial of sugar water. If no discomfort occurred within 6 h, the diet was changed to semi-liquid, and gradually restored to regular food after 24 h. A high-protein, high-fiber diet was recommended to promote recovery.

### 2.3 Outcome measures

#### 2.3.1 Neurological function

Neurological status was evaluated using the National Institutes of Health Stroke Scale (NIHSS) (13) at baseline (preoperative) and 1 month postoperatively. This 11-item scale assesses domains including consciousness, sensory function, and inattention, with total scores ranging from 0 to 42. Higher scores indicate greater neurological impairment.

#### 2.3.2 Limb motor function

Limb motor function was measured through the Fugl-Meyer Assessment (FMA) (14) preoperatively and at 1-month follow-up. The scale comprises 33 items for upper limbs (scored 0–66) and 17 items for lower limbs (scored 0–34), yielding a total score of 0–100, where higher scores reflect better motor performance.

#### 2.3.3 QoL

The 36-Item Short-Form Health Survey (SF-36) (15) was administered preoperatively and 1-month postoperatively to assess QoL across eight domains: physical functioning, bodily pain, role-physical, general health, social functioning, vitality, role-emotional, and mental health. Each domain is scored from 0 to 100, with higher scores denoting better QoL.

#### 2.3.4 Postoperative complications

Cranial computed tomography (CT) scans were performed monthly for 6 months postoperatively to monitor complications, including pneumocephalus, surgical site infection, and subdural effusion.

#### 2.3.5 Recurrence criteria

Recurrence of CSDH was defined as new-onset neurological deficits (e.g., altered consciousness, hemiparesis, dizziness, or headache) within 6 months postoperatively after excluding other neurological etiologies. Patients were stratified into recurrence and non-recurrence groups based on these criteria.

### 2.4 Statistical analysis

All statistical analyses were performed using SPSS 26.0 software (IBM Corp., Armonk, NY, United States). Continuous variables were assessed for normality using the Shapiro–Wilk test and expressed as mean ± standard deviation ( $\bar{x}$ ± s) if normally distributed, with between-group comparisons conducted via independent samples *t*-test. Categorical variables were summarized as *n* (%), with group differences analyzed using the Chi-square (*x*^2^) test or Fisher’s exact test when expected cell frequencies were <5. To identify factors influencing postoperative recurrence, clinical variables were first screened through univariate analysis, retaining those with significant associations (*p* < 0.05). These variables were subsequently entered into a multivariate logistic regression model to establish a predictive model and determine independent risk factors for recurrence. ROC curves were plotted to evaluate the predictive performance of relevant indicators, with calculation of the area under the curve (AUC), sensitivity, specificity, and optimal cut-off values to assess their clinical diagnostic utility. Statistical significance was defined as *p* < 0.05 for all analyses.

## 3 Results

### 3.1 Comparison of baseline characteristics between groups

No statistically significant differences were observed between the control and observation groups in demographic or clinical characteristics, including gender, age, preoperative diabetes mellitus, hypertension, trauma history, hematoma laterality, brain atrophy, brain reexpansion status, preoperative hematoma width/density, midline shift, and postoperative subdural space (all *p* > 0.05), suggesting comparability of baseline characteristics between the two groups. Details are presented in Table 1.

**Table 1:** Comparison of baseline characteristics between groups [ $\bar{x}$ ± *s*, *n* (%)].

Characteristic		Control group *n* = 92	Observation group *n* = 92	*x*^2^/*t*	*P*
Age (years)		61.17 ± 4.52	61.73 ± 4.77	0.83	0.41
Gender	Male	67 (72.83%)	61 (66.3%)	0.92	0.34
	Female	25 (27.17%)	31 (33.7%)		
History of diabetes mellitus	Yes	12 (13.04%)	15 (16.3%)	0.39	0.53
	No	80 (86.96%)	77 (83.7%)		
History of hypertension	Yes	15 (16.3%)	18 (19.57%)	0.33	0.56
	No	77 (83.7%)	74 (80.43%)		
Trauma history	Yes	67 (72.83%)	62 (67.39%)	0.65	0.42
	No	25 (27.17%)	30 (32.61%)		
Hematoma laterality	Left	44 (47.83%)	38 (41.3%)	0.89	0.64
	Right	36 (39.13%)	39 (42.39%)		
	Bilateral	12 (13.04%)	15 (16.3%)		
Brain atrophy	Yes	42 (45.65%)	48 (52.17%)	0.78	0.38
	No	50 (54.35%)	44 (47.83%)		
Brain reexpansion status	Good	69 (75.0%)	64 (69.57%)	0.68	0.41
	Poor	23 (25.0%)	28 (30.43%)		
Preoperative hematoma width (mm)	≥20	54 (58.7%)	49 (53.26%)	0.55	0.46
	<20	38 (41.3%)	43 (46.74%)		
Preoperative hematoma density	Low density	63 (68.48%)	55 (59.78%)	1.81	0.41
	Mixed density	27 (29.35%)	33 (35.87%)		
	High density	2 (2.17%)	4 (4.35%)		
Preoperative midline shift (mm)	≥10	61 (66.3%)	66 (71.74%)	0.64	0.43
	<10	31 (33.7%)	26 (28.26%)		
Postoperative subdural space (mm)	≥10	24 (26.09%)	21 (22.83%)	0.27	0.61
	<10	68 (73.91%)	71 (77.17%)		

### 3.2 Comparison of postoperative neurological recovery between groups

No significant differences in preoperative neurological assessments were observed between the groups, as evidenced by comparable NIHSS scores (*t* = 0.51, *p* = 0.61) and FMA scores (*t* = 1.48, *p* = 0.14). At 1-month follow-up, both groups exhibited neurological improvement compared to baseline. However, the observation group demonstrated superior recovery outcomes, with significantly lower NIHSS scores (*t* = 4.94, *p* < 0.001) and higher FMA scores (*t* = 3.77, *p* < 0.001) than the control group (Table 2).

**Table 2:** Comparison of postoperative neurological recovery between groups [ $\bar{x}$ ± *s*].

Group	NIHSS score		FMA score	
	Before surgery	1 month after surgery	Before surgery	1 month after surgery
Control	34.14 ± 4.01	27.35 ± 3.05	58.63 ± 5.62	68.59 ± 3.99
Observation	33.82 ± 4.57	24.97 ± 3.47	59.83 ± 5.32	71.21 ± 5.33
*t*	0.51	4.94	1.48	3.77
*P*	0.61	<0.001	0.14	<0.001

NIHSS, the National Institutes of Health Stroke Scale; FMA, the Fugl-Meyer Assessment.

### 3.3 Comparison of postoperative QoL between groups

Preoperatively, no significant differences were found in any SF-36 domain scores between the two groups (*p* > 0.05), demonstrating comparable baseline profiles. At 1-month postoperatively, the observation group exhibited significantly higher scores than the control group across all eight domains, including physical functioning, bodily pain, role-physical, general health, social functioning, vitality, role-emotional, and mental health (all *p* < 0.01), as displayed in Table 3.

**Table 3:** Comparison of postoperative QoL between groups [ $\bar{x}$ ± *s*].

Group	Physical functioning		Bodily pain	
	Before surgery	1 month after surgery	Before surgery	1 month after surgery
Control	46.13 ± 5.45	63.24 ± 5.40	50.42 ± 4.80	71.29 ± 4.66
Observation	45.34 ± 4.97	70.95 ± 5.51	50.94 ± 5.07	74.05 ± 5.28
*t*	1.03	9.58	0.72	3.76
*P*	0.30	<0.001	0.48	<0.001

Group	Role-physical		General health	
	Before surgery	1 month after surgery	Before surgery	1 month after surgery
Control	52.45 ± 5.68	69.04 ± 5.32	52.74 ± 4.18	64.92 ± 5.80
Observation	52.34 ± 5.28	72.94 ± 4.66	52.32 ± 4.10	71.12 ± 5.75
*t*	0.13	5.27	0.69	7.28
*P*	0.89	<0.001	0.49	<0.001

Group	Social functioning		Vitality	
	Before surgery	1 month after surgery	Before surgery	1 month after surgery
Control	50.39 ± 5.17	70.48 ± 4.64	51.64 ± 5.10	70.79 ± 5.94
Observation	50.64 ± 5.38	72.55 ± 4.87	51.32 ± 4.76	75.65 ± 5.49
*t*	0.32	2.96	0.45	5.76
*P*	0.75	0.003	0.66	<0.001

Group	Role-emotional		Mental health	
	Before surgery	1 month after surgery	Before surgery	1 month after surgery
Control	58.11 ± 6.47	77.24 ± 6.31	51.02 ± 5.01	75.27 ± 6.44
Observation	58.35 ± 6.43	84.09 ± 5.30	51.56 ± 5.78	79.09 ± 5.11
*t*	0.25	7.97	1.05	4.45
*P*	0.80	<0.001	0.30	<0.001

### 3.4 Comparison of postoperative complications and recurrence between groups

Overall postoperative complication incidence and CSDH recurrence rate were significantly lower in the observation group compared to the control group (*p* < 0.05). Specifically, the observation group exhibited reduced incidences of pneumocephalus (1.09% vs. 4.35%), surgical site infection (0% vs. 3.26%), and subdural effusion (1.09% vs. 3.26%). The 6-month CSDH recurrence rate was also markedly lower in the observation group (7.61% vs. 19.57%), as shown in Table 4.

**Table 4:** Comparison of postoperative complications and recurrence between groups [*n* (%)].

Group	Complication				Recurrence rate
	Pneumocephalus	Surgical site infection	Subdural effusion	Overall incidence	
Control	4 (4.35%)	3 (3.26%)	3 (3.26%)	10 (10.87%)	18 (19.57%)
Observation	1 (1.09%)	0 (0.00%)	1 (1.09%)	2 (2.17%)	7 (7.61%)
*x*^2^	5.71				5.60
*P*	0.02				0.02

### 3.5 Univariate analysis of clinical factors influencing postoperative recurrence

Univariate analysis identified several clinical factors significantly correlated with postoperative CSDH recurrence. Younger patient age (*t* = 2.73, *p* = 0.019), brain atrophy (*x*^2^ = 4.22, *p* = 0.04), poor brain reexpansion (*x*^2^ = 5.94, *p* = 0.015), preoperative hematoma width ≥ 20 mm (*x*^2^ = 4.69, *p* = 0.03), preoperative hematoma density distribution (Fisher’s exact test, *p* = 0.005), and postoperative subdural space ≥ 10 mm (*x*^2^ = 5.98, *p* = 0.014) were significant predictors of recurrence. Notably, nursing interventions incorporating MPNFS combined with HBOT demonstrated protective effects (*x*^2^ = 10.42, *p* = 0.001). No significant correlations were observed for gender, diabetes mellitus, hypertension, trauma history, hematoma laterality, or midline shift (all *p* > 0.05) (Table 5).

**Table 5:** Univariate analysis of clinical factors influencing postoperative recurrence [ $\bar{x}$ ± *s*, *n* (%)].

Characteristic		Recurrence group *n* = 25	Non-recurrence group *n* = 159	*x*^2^/t	*P*
Age (years)		61.14 ± 4.52	63.48 ± 5.01	2.73	0.019
Gender	Male	17 (68.0%)	111 (69.81%)	0.03	0.87
	Female	8 (32.0%)	48 (30.19%)		
Preoperative diabetes mellitus	Yes	5 (20.0%)	23 (14.47%)	0.51	0.47
	No	20 (80.0%)	136 (85.53%)		
Preoperative hypertension	Yes	7 (28.0%)	26 (16.35%)	1.99	0.16
	No	18 (72.0%)	133 (83.65%)		
Trauma history	Yes	10 (40.0%)	45 (28.3%)	1.41	0.24
	No	15 (60.0%)	114 (71.7%)		
Hematoma laterality	Left	10 (40.0%)	72 (45.28%)	0.70	0.71
	Right	10 (40.0%)	65 (40.88%)		
	Bilateral	5 (20.0%)	22 (13.84%)		
Brain atrophy	Yes	17 (68.0%)	73 (45.91%)	4.22	0.04
	No	8 (32.0%)	86 (54.09%)		
Brain reexpansion status	Good	13 (52.0%)	120 (75.47%)	5.94	0.015
	Poor	12 (48.0%)	39 (24.53%)		
Preoperative hematoma width (mm)	≥20	16 (64.0%)	65 (40.88%)	4.69	0.03
	<20	9 (36.0%)	94 (59.12%)		
Preoperative hematoma density	Low density	13 (52.0%)	105 (66.04%)	15.07	Fisher = 0.005
	Mixed density	8 (32.0%)	52 (32.7%)		
	High density	4 (16.0%)	2 (1.26%)		
Preoperative midline shift (mm)	≥10	21 (84.0%)	106 (66.67%)	3.04	Fisher = 0.10
	<10	4 (16.0%)	53 (33.33%)		
Postoperative subdural space (mm)	≥10	11 (44.0%)	34 (21.38%)	5.98	0.014
	<10	14 (56.0%)	125 (78.62%)		
Nursing intervention	Routine	5 (20.0%)	87 (54.72%)	10.42	0.001
	MPNFS + HBOT	20 (80.0%)	72 (45.28%)		

MPNFS, Medication, Psychological intervention, Nursing, Family care, and Social support; HBOT, hyperbaric oxygen therapy.

### 3.6 Multivariate logistic regression analysis of independent risk factors for CSDH recurrence

Using postoperative CSDH recurrence as the dependent variable, and variables with *p* < 0.05 from Table 5 as independent variables (variable coding details in Table 6), a multivariate logistic regression model was constructed. The analysis identified four independent risk factors of recurrence: brain atrophy (OR = 2.877, *p* = 0.043), inadequate brain reexpansion (OR = 3.165, *p* = 0.025), preoperative hematoma width ≥ 20 mm (OR = 2.782, *p* = 0.040), and routine nursing care (vs. MPNFS + HBOT, OR = 2.842, *p* = 0.049). Complete regression results are seen in Tables 6, 7.

**Table 6:** Variable coding for CSDH recurrence analysis.

Variable	Assignment criteria
Recurrence	Non-recurrence = 1, Recurrence = 2
Brain atrophy	No = 0, Yes = 1
Brain reexpansion status	Good = 1, Poor = 2
Preoperative hematoma width	<20 mm = 0, ≥20 mm = 1
Preoperative hematoma density	Low density = 1, Mixed density = 2, High density = 3
Postoperative subdural space	< 10 mm = 0, ≥ 10 mm = 1
Nursing intervention	Routine care = 1, MPNFS + HBOT = 2

MPNFS, Medication, Psychological intervention, Nursing, Family care, and Social support; HBOT, hyperbaric oxygen therapy.

**Table 7:** Multivariate logistic regression analysis of independent risk factors for CSDH recurrence.

Index	*β*	SE	Wald *x*^2^	*P*	Exp (B)	95%CI
Constant	−10.077	3.439	8.588	0.003	0	–
Age	0.085	0.054	2.529	0.112	1.089	0.98–1.21
Brain atrophy	1.07	0.523	4.186	0.041	2.915	1.046–8.121
Brain reexpansion status	1.09	0.517	4.45	0.035	2.975	1.08–8.194
Preoperative hematoma width	1.048	0.505	4.302	0.038	2.853	1.059–7.683
Preoperative hematoma density	–	–	2.318	0.314	–	–
Preoperative hematoma density (1)	0.541	0.536	1.018	0.313	1.717	0.601–4.911
Preoperative hematoma density (2)	1.22	0.918	1.764	0.184	3.387	0.56–20.487
Postoperative subdural space	0.879	0.539	2.66	0.103	2.41	0.837–6.933
Nursing intervention	1.294	0.563	5.288	0.021	3.647	1.211–10.987

### 3.7 ROC analysis of predictive value for postoperative recurrence

**Figure 1:**
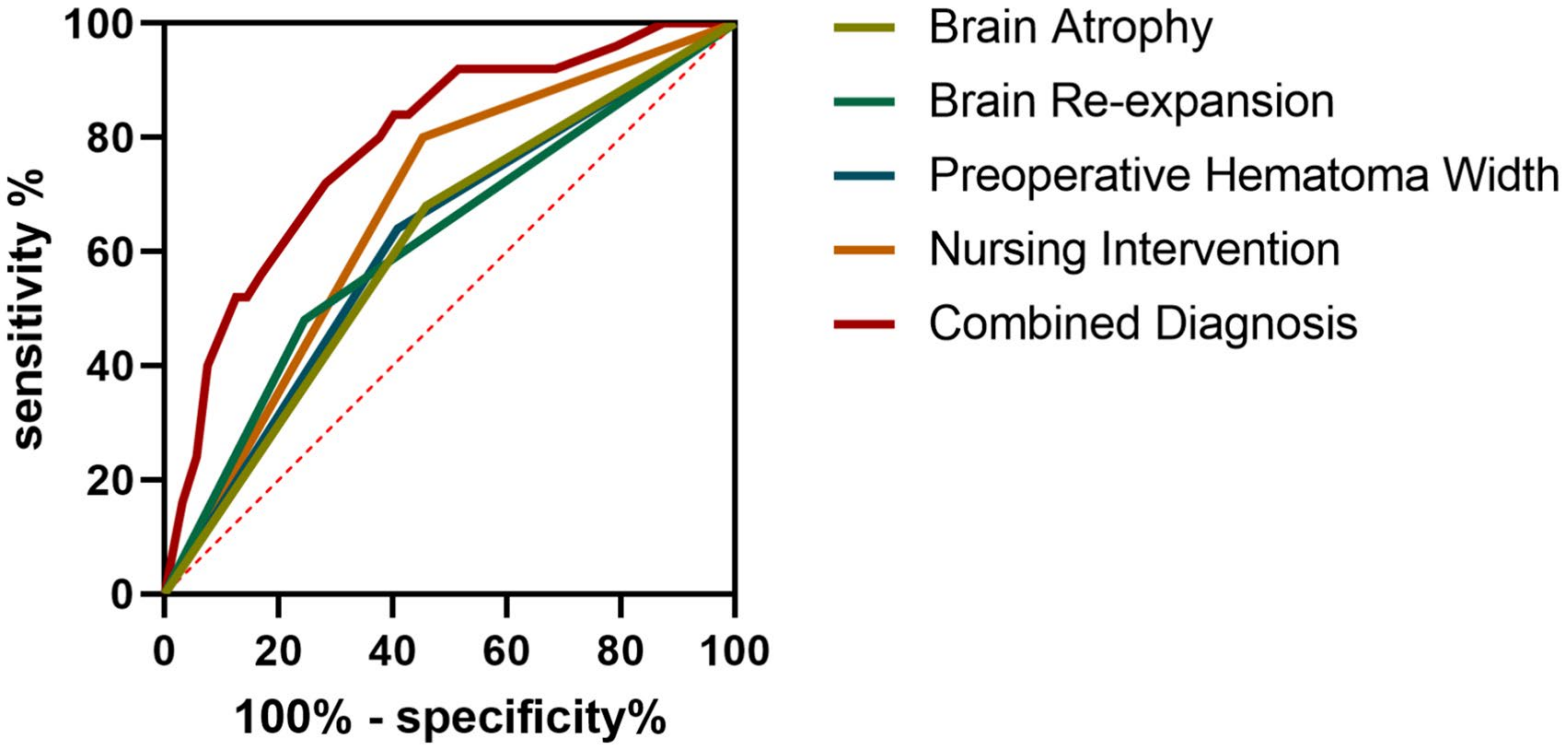
Receiver operating characteristic (ROC) curves for postoperative recurrence prediction.

To further evaluate the discriminative capacity of key predictors (brain atrophy, poor brain reexpansion, preoperative hematoma width ≥ 20 mm, and routine nursing care) and their combination for recurrence risk, ROC curve analysis was performed. Individual predictors including brain atrophy, brain reexpansion status, preoperative hematoma width, and nursing intervention demonstrated moderate predictive utility, with nursing intervention exhibiting the highest AUC (0.6736) and sensitivity (80.0%). The multivariable predictive model integrating these factors showed enhanced performance, achieving an AUC of 0.7862 with 84% sensitivity and 59.75% specificity, indicating robust diagnostic performance in recurrence risk stratification (Table 8 and Figure 1).

**Table 8:** Diagnostic performance of recurrence predictors.

Predictor	AUC	Sensitivity%	Specificity%	Cutoff	95% CI
Brain atrophy	0.6104	68%	54.09%	0.2209	0.4942–0.7267
Brain re-expansion	0.6174	48%	75.47%	0.2347	0.4930–0.7417
Preoperative hematoma width	0.6156	64%	59.12%	0.2312	0.4979–0.7333
Nursing intervention	0.6736	80%	54.72%	0.3472	0.5677–0.7795
Combined diagnosis	0.7862	84%	59.75%	0.4375	0.6924–0.8799

AUC, the area under the curve.

## 4 Discussion

This study evaluated the role of MPNFS model combined with HBOT in the rehabilitation of CSDH patients after burr hole drainage, aiming to systematically assess the synergistic effects of this combined intervention on neurological function, limb motor function, QoL, postoperative complications, and recurrence risk. The results showed that the observation group outperformed the control group in all measured indices, indicating that the combined intervention significantly promotes postoperative recovery, improves QoL, and reduces complications and recurrence risk, with promising clinical application potential.

In neurological recovery, the observation group demonstrated superior outcomes over the control group, with significantly lower NIHSS scores at 1-month follow-up, directly evidencing reduced neurological deficits. HBOT primes a favorable physiological foundation for neural restoration by enhancing oxygen perfusion and stimulating metabolic repair of ischemic brain tissue (16). Concurrently, MPNFS nursing interventions, via personalized health education, psychological support, and early mobility guidance, facilitate mental adjustment, build rehabilitation confidence, and enhance patient engagement, thereby accelerating functional neurological improvement in tandem with HBOT’s biological effects (17). Supplementary measures such as postural management, structured motor training, and emotional counseling further foster multifaceted brain function reconstruction while mitigating secondary injuries associated with prolonged immobility (18). In terms of motor function, the observation group exhibited significantly elevated FMA scores relative to the control group, suggesting that, under equivalent conditions of HBOT and basic mobility guidance, the structured and individualized rehabilitation planning within the MPNFS model may offer additional benefits. This model promotes sustained motor recovery by enhancing patient motivation and adherence through continuous health education, goal setting, process monitoring, and active family involvement, thereby helping to reduce disuse-related muscle atrophy and joint stiffness (19). Previous research also supports this interpretation: psychosocial interventions led by nurses—including structured education, follow-up, and peer support—have been shown to enhance stroke survivors’ self-management and engagement, ultimately improving functional outcomes and HRQoL (20).

With regard to quality of life, the intervention group also exhibited significantly higher SF-36 scores at 1-month postoperatively. Given that both groups received the same HBOT protocol, the observed intergroup differences are more likely attributable to the psychosocial and self-management components of the MPNFS model. These include structured health education, emotional support, goal-oriented home care guidance, and the involvement of family members and peers—all of which help promote patient initiative and treatment adherence, contributing to HRQoL improvement. Findings from randomized controlled trials and systematic reviews support this view, indicating that nurse-led education and follow-up programs, peer support, and web-based interventions can significantly improve HRQoL among stroke survivors and reduce caregiver burden (21, 22).

As for postoperative complications, the observation group had a significantly lower incidence of complications within 6 months postoperatively compared to the control group. This disparity was particularly pronounced in common complications such as pneumocephalus, surgical site infection, and subdural effusion (23), where the observation group’s incidence rates were notably reduced. The MPNFS nursing model achieved this through meticulous interventions in drainage tube management, individualized nutritional support, and early mobilization protocols, which were designed to minimize iatrogenic risks and promote physiological homeostasis. Simultaneously, HBOT provided biological protection against complications by reducing cerebral edema, accelerating hematoma resorption, and inhibiting inflammatory responses, thereby creating a favorable microenvironment for postsurgical recovery.

Another critical focus of this study was recurrence risk. Postoperative 6-month follow-up and imaging evaluations revealed that the observation group showed a substantially lower recurrence rate than the control group. Multivariate logistic regression analysis identified brain atrophy, poor brain reexpansion, and preoperative hematoma width ≥ 20 mm as independent risk factors for recurrence. Mechanistically, brain atrophy, characterized by degenerative reduction in cerebral volume, impedes postoperative brain reexpansion, increasing the likelihood of residual hematoma and rebleeding. Poor brain reexpansion, in turn, may disrupt local neurological homeostasis, creating abnormal pressure gradients that predispose to hematoma recurrence. Patients with larger preoperative hematomas face severer initial lesions and greater postoperative recovery challenges, inherently elevating their recurrence risks. ROC curve analysis further indicated that the developed recurrence prediction model exhibited robust discriminative ability, providing a clinical reference for screening high-risk individuals and tailoring personalized preventive interventions.

While this study yielded valuable insights, several limitations need acknowledgment. First, this study employed a combined intervention approach in which the intervention group received both HBOT and nursing care based on the MPNFS model. As no separate comparator groups were established for each component, it is difficult to determine the individual contribution of each intervention to the observed outcomes. This limitation may affect the interpretability of the underlying mechanisms. Future research should consider using factorial designs or multi-arm trials to further clarify the relative effects of each component. Second, the relatively small sample size and single-center design, enrolling patients from a single geographical region, restrict the generalizability of these findings, as the results may not fully apply to more diverse populations. Thus, future multicenter trials with larger cohorts are essential to validate the current conclusions and enhance external validity. Second, the 6-month follow-up period is insufficient to evaluate the long-term effects of the combined HBOT-MPNFS intervention. Fourth, although this study employed instruments such as the NIHSS, FMA, and SF-36 to assess postoperative recovery, it did not include measures such as the modified Rankin Scale (mRS), Functional Independence Measure (FIM), or Barthel Index, which are more direct indicators of functional independence. The absence of these tools may have limited the granularity of assessment regarding self-care ability. Future studies should incorporate a more comprehensive evaluation framework to better capture patients’ functional and social reintegration outcomes. In addition, process-related variables such as patient adherence, family involvement, and subjective rehabilitation experience were not systematically evaluated. These areas represent valuable directions for future research in nursing-based interventions. Lastly, this study focused primarily on clinical efficacy and did not include neurobiological or imaging-based indicators. Further research incorporating neuroimaging, biochemical markers, and psychological assessments may help elucidate the mechanisms underlying the combined intervention.

In conclusion, the combination of the MPNFS-based nursing model and hyperbaric oxygen therapy demonstrated favorable short-term clinical benefits in patients undergoing rehabilitation after CSDH surgery. Future investigations should prioritize expanding sample sizes, extending follow-up periods, and conducting multicenter trials to comprehensively evaluate the long-term efficacy and underlying mechanisms of this combined approach. Such efforts will facilitate its broader implementation and promotion in neurosurgical nursing practice, ultimately translating research insights into improved patient care.

## 5 Conclusion

The MPNFS nursing model combined with HBOT can significantly promote neurological recovery, improve limb motor function, enhance QoL, and effectively reduce postoperative complications and recurrence risk in patients after CSDH surgery. This integrated approach holds promising clinical potential, offering a more effective therapeutic option for rehabilitation among such patients.

## Data Availability

The original contributions presented in the study are included in the article/supplementary material, further inquiries can be directed to the corresponding author.
